# Practical approach to thrombocytopenia in patients with sepsis: a narrative review

**DOI:** 10.1186/s12959-024-00637-0

**Published:** 2024-07-22

**Authors:** Kasumi Satoh, Takeshi Wada, Akihito Tampo, Gaku Takahashi, Kota Hoshino, Hironori Matsumoto, Takayuki Taira, Satoshi Kazuma, Takamitsu Masuda, Takashi Tagami, Hiroyasu Ishikura, J-STAD (JAPAN Sepsis Treatment and Diagnosis) Study Group, Takayuki Ogura, Yu Kawazoe, Yudai Takatani, Chie Tanaka, Kensuke Nakamura, Yoshihiko Nakamura, Katsunori Mochizuki, Maiko Yamazaki

**Affiliations:** 1 Advanced Emergency and Critical Care Center, Akita University Hospital, Akita, Japan; 2 Division of Acute and Critical Care Medicine, Department of Anesthesiology and Critical Care Medicine, Hokkaido University Faculty of Medicine, Kita 15, Nishi 7, Kita-ku, Sapporo, 060-8638 Japan; 3 Department of Emergency Medicine, Asahikawa Medical University, Asahikawa, Japan; 4 Department of Critical Care, Disaster and General Medicine, School of Medicine, Iwate Medical University, Iwate, Japan; 5 Department of Emergency and Critical Care Medicine, Faculty of Medicine, Fukuoka University, Fukuoka, Japan; 6 Department of Emergency and Critical Care Medicine, Ehime University Graduate School of Medicine, Toon, Japan; 7 Department of Emergency and Critical Care Medicine, Graduate School of Medicine, University of the Ryukyus, Okinawa, Japan; 8 Department of Intensive Care Medicine, School of Medicine, Sapporo Medical University, Sapporo, Hokkaido Japan; 9 Department of Emergency Medicine, Emergency and Critical Care Center, Fujieda Municipal General Hospital, Fujieda, Japan; 10 Department of Emergency and Critical Care Medicine, Nippon Medical School Musashikosugi Hospital, Tokyo, Japan

**Keywords:** Intensive Care Units (ICU), Disseminated Intravascular Coagulation (DIC), Thrombotic Microangiopathy (TMA), Thrombotic Thrombocytopenic Purpura (TTP), Anticoagulant therapy, Hemolytic Uremic Syndrome (HUS), Hemolytic Anemia, a disintegrin-like and metalloproteinase with thrombospondin type 1 motifs 13 (ADAMTS13)

## Abstract

Thrombocytopenia frequently occurs in patients with sepsis. Disseminated intravascular coagulation (DIC) may be a possible cause of thrombocytopenia owing to its high prevalence and association with poor outcomes; however, it is important to keep the presence of other diseases in mind in sepsis practice. Thrombotic microangiopathy (TMA), which is characterized by thrombotic thrombocytopenic purpura, Shiga toxin-producing Escherichia coli hemolytic uremic syndrome (HUS), and complement-mediated HUS, is characterized by thrombocytopenia, microangiopathic hemolytic anemia, and organ damage. TMA has become widely recognized in recent years because of the development of specific treatments. Previous studies have reported a remarkably lower prevalence of TMA than DIC; however, its epidemiology is not well defined, and there may be cases in which TMA is not correctly diagnosed, resulting in poor outcomes. Therefore, it is important to differentiate DIC from TMA. Nevertheless, differentiating between DIC and TMA remains a challenge as indicated by previous reports that most patients with TMA can be diagnosed as DIC using the universal coagulation scoring system. Several algorithms to differentiate sepsis-related DIC from TMA have been suggested, contributing to improving the care of septic patients with thrombocytopenia; however, it may be difficult to apply these algorithms to patients with coexisting DIC and TMA, which has recently been reported. This review describes the disease characteristics, including epidemiology, pathophysiology, and treatment, of DIC, TMA, and other diseases with thrombocytopenia and proposes a novel practical approach flow, which is characterized by the initiation of the diagnosis of TMA in parallel with the diagnosis of DIC. This practical flow also refers to the longitudinal diagnosis and treatment flow with TMA in mind and real clinical timeframes. In conclusion, we aim to widely disseminate the results of this review that emphasize the importance of incorporating consideration of TMA in the management of septic DIC. We anticipate that this practical new approach for the diagnostic and treatment flow will lead to the appropriate diagnosis and treatment of complex cases, improve patient outcomes, and generate new epidemiological evidence regarding TMA.

## Background

Thrombocytopenia is a relatively common complication in patients with sepsis. In septic disseminated intravascular coagulation (DIC), marked coagulation activation causes multiple microfibrin thrombi within the systemic microvasculature. As the condition progresses, platelets are consumed along with coagulation factors, resulting in thrombocytopenia [1, 2]. In addition, recent studies have revealed that platelet activation occurs along with coagulation activation, and that platelets activated together with neutrophils accumulate in the lungs and liver, resulting in a decrease in platelets [3, 4]. Owing to these effects, septic DIC is often complicated by thrombocytopenia. However, immunothrombosis is the primary underlying cause of thrombocytopenia in patients with infectious diseases, which plays a physiological role in preventing systemic pathogen dissemination Moreover, not all sepsis cases result in pathological coagulopathy, namely, DIC. Therefore, we should note that thrombocytopenia may occur in patients with sepsis even in conditions other than DIC. Thrombotic microangiopathy (TMA) is pathologically diagnosed based on three symptoms: thrombocytopenia, microangiopathic hemolytic anemia (MAHA), and organ damage [5]. It is a relatively rare disease with a potentially poor prognosis without early intervention, and is difficult to differentiate from DIC [6]. Azoulay et al. reported that although physicians examine an average of three TMA patients per year in the intensive care unit (ICU), a number of them are incorrectly diagnosed upon admission [7].

Many clinicians attribute sepsis-induced DIC as the cause of thrombocytopenia in ICU patients; upon examining the infection, the use of antibiotics will be subsequently considered. If the condition is severe, platelet transfusion, heparin administration, and, in some countries including Japan, recombinant human thrombomodulin (rhTM) or antithrombin treatment may be considered [8]. However, improving TMA using these treatments remains difficult. Although rare, TMA may coexist with septic DIC. If TMA cannot be identified, this may result to poor patient outcomes.

This review outlined the epidemiology and pathophysiology of DIC. Subsequently, we explored TMA, which is a representative condition of septic thrombocytopenia without DIC. We compared the similarities and differences between DIC and TMA and proposed a new practical approach to simultaneously diagnose these two conditions. Additionally, thrombocytopenia is associated with various severe diseases and pathological conditions other than TMA and DIC. These include hemophagocytic lymphohistiocytosis (HLH), characterized by excessive inflammatory responses; emerging infectious diseases such as coronavirus disease 2019 (COVID-19); chronic conditions such as liver cirrhosis; and treatment-related complications such as heparin-induced thrombocytopenia (HIT). Although these diseases and conditions cause thrombocytopenia through various mechanisms, they are commonly encountered in the intensive care unit, manifesting alongside or mimicking sepsis, and significantly affect patient outcomes. This article provides a comprehensively review of these diseases and their associated pathological conditions.

### Epidemiology

Thrombocytopenia is a common disorder in patients requiring intensive care, which account for 25–55% of patients admitted to the ICU [9] and 50% of those admitted to the ICU after surgery [10]. Thrombocytopenia can be attributed to DIC, and TMA including thrombotic thrombocytopenic purpura (TTP), and hemolytic uremic syndrome (HUS); of which, DIC is the most common, with sepsis-associated DIC reported at 9–19% of thrombocytopenia cases [11]. Although many studies on thrombocytopenia have been published, reports on the incidence of thrombocytopenia due to TMA, are scarce.

In previous studies, the incidence of thrombocytopenia in patients with sepsis ranged from 37.5–83.5% [12–18]. However, most of the studies used data from a single or two centers, and TMA was diagnosed in only one study. The number of patients with sepsis and thrombocytopenia due to DIC and TMA reported in previously published papers is summarized in Table 1. Of the eight papers, two were on neonates or children, and six were on adults. Among the eight studies reporting a case of thrombocytopenia in patients with sepsis, five attributed thrombocytopenia to DIC and one to TMA. Sevketoglu et al. [19] reported a diagnosis of TTP; however, the total number of patients with sepsis was unknown as the population comprised patients hospitalized for thrombocytopenia with sepsis. Notably, only one of the eight studies reported thrombocytopenia due to both DIC and TMA.

**Table 1:** Number of patients with sepsis, thrombocytopenia, disseminated intravascular coagulation, and thrombotic microangiopathy in reviewed articles

**Author**	**Participants**	**Sepsis**	**Thrombocytopenia**	**DIC**	**TMA**
Claushuis et al. [12]	Adults	929	349 (37.5%)	No data	No data
Ree et al. [13]	Adults	460	226 (49%)	No data	No data
Venkata et al. [14]	Adults	304	145 (47.6%)	37 (25.5%)	No data
Li et al. [15]	Adults	261	127 (48.6%)	No data	No data
Bedet et al. [16]	Adults	60	33 (55%)	31 (51%)	No data
Abe et al. [17]	Adults	49	No data	34 (69%)	No data
Arif et al. [18]	Neonates	85	71 (83.5%)	5 (5.8%)	No data
Sevketoglu et al. [19]	Childs	No data	42	27	4 (9% TTP)

Data are expressed as n (%).

*DIC* Disseminated intravascular coagulation, *TMA* Thrombotic microangiopathy, *TTP* Thrombotic thrombocytopenic purpura

## DIC

### Pathology

DIC is characterized by intravascular activation of coagulation and excessive thrombin generation, which deteriorates with the impairment of anticoagulant pathways, resulting in simultaneous widespread microvascular thrombosis, leading to organ ischemia and multiple organ dysfunction [20–22]. This subsequently consumes platelets and coagulation factors, leading to severe bleeding at various sites [23]. DIC is subdivided into fibrinolytic and hypofibrinolytic phenotypes [24]. In the fibrinolytic phenotype, which occurs during trauma in the early phase and hematological malignancies, the underlying disease of DIC induces excessive hyperfibrinolysis by a mechanism other than secondary fibrinolysis to hypercoagulation, contributing to massive bleeding [25]. Sepsis is a representative disease that develops in the hypofibrinolytic phenotype, in which the plasminogen activator inhibitor-1 (PAI-1) induced by inflammation suppresses fibrinolysis, further aggravating ischemic organ dysfunction [26]. The concept that excessive immunothrombosis-forming intravascular thrombi can lead to DIC has been established recently [27]. Immunothrombosis has some functions on host defense, including pathogen recognition, pathogen compartmentalization and trapping, prevention of pathogen spreading and invasion by forming microvascular thrombus, and activated neutrophil extracellular traps (NETs), which play important roles in immunothrombosis formation [27]. Histones, the main component of NETs, promote further NETs release. NETs and histones have been shown to induce the pathomechanisms of DIC, including procoagulant, inflammatory, hypofibrinolytic, and cytotoxic effects, and DIC is viewed as a dysregulated inflammatory coagulofibrinolytic response resulting from the synergistic effects of NETs and histones [28, 29]. Immunothrombosis also indicates the pathways for the activation of platelets [27]. Platelet activation involves the hemostatic and immune receptor-mediated pathways as the cells’ initial response to infection. The hemostatic receptors, Glycoprotein (GP) IIb/IIIa and GPIb, are involved in platelet activation by interacting with the fibrinogen and von Willebrand factor (vWF), respectively. The immunoreceptor FcγRIIa interacts with pathogen-bound immunoglobulin G, and toll-like receptors interact with pathogens, both of which play important roles in platelet aggregation [30].

### DIC diagnosis

An accurate diagnosis of DIC requires evidence of the presence of an intravascular fibrin clot; however, this is not clinically realistic. As there is no gold standard for DIC diagnosis, the use of scoring systems is recommended [31]. Sets of DIC diagnostic criteria have been proposed by the International Society on Thrombosis and Haemostasis (ISTH) [22], the Japanese Ministry of Health and Welfare (JMHW) [32], and the Japanese Association of Acute Medicine (JAAM) [33], which consist of several coagulation-fibrinolysis markers. The latest set of DIC criteria was proposed by the Japanese Society on Thrombosis and Hemostasis (JSTH) [34, 35] (Table 2). Although there is no gold standard for DIC diagnosis, the DIC scoring system plays an important role in selecting patients who require treatment for coagulation-fibrinolysis abnormalities [36].

**Table 2:** Criteria for diagnosing DIC

	**ISTH** [22]		**JMHW** [32]		**JAAM** [33]		**JSTH (infection type)** [34, 35]	
**SIRS symptoms**	-		-		≥3	1	-	
**Etiology**	Require		(+)	1	-		-	
**Bleeding symptom**	-		(+)	1	-		-	
**Organ dysfunction**	-		(+)	1	-		Severe liver dysfunction	-3
**Platelet count (10**^**3**^** /µl)**	50≤, ≤100	1	80<, ≤120	1	80≤, <120	1	80<, ≤120	1
	<50	2	50<, ≤80	2	<80	3	50<, ≤80	2
	-		≤50	3	-		≤50	3
**PTINR**	-		1.25≤, <1.67	1	≥1.2	1	1.25≤, <1.67	1
			≥1.67	2			≥1.67	2
**PT (sec)**	3<, <6	1	-		-		-	
	>6	2						
**Fibrinogen (µg/ml)**	<100	1	100<, ≤150	1	-		-	
			≤100	2				
**FDP (µg/ml)**	Moderate increase	2	10 ≤ , <20	1	10≤, <25	1	10 ≤ , <20	1
	Strong increase	3	20≤ , <40	2	≥25	3	20≤ , <40	2
	-		≥40	3	-		≥40	3
**Antithrombin (%)**	-		-		-		≤70	1
**TAT, SF, F1+2**	-		-		-		≥ 2-fold of normal upper limit	1
**DIC diagnosis**	**≥5 points**		**≥7 points**		**≥4 points**		**≥5 points**	

*DIC* Disseminated intravascular coagulation, *JMHW* Japanese Ministry of Health and Welfare, *ISTH* International Society on Thrombosis and Haemostasis, *JAAM* Japanese Association of Acute Medicine, *JSTH* Japanese Society on Thrombosis and Hemostasis, *SIRS* Systemic Inflammatory Response Syndrome, *PT* Prothrombin time, *PT-INR* Prothrombin time-international normalized ratio, *FDP* Fibrin degradation product, *TAT* Thrombin-antithrombin complex, *SF* Soluble fibrin, *F1+2* Prothrombin fragment

The two sets of DIC criteria were composed of similar coagulation-fibrinolysis markers as the ISTH DIC criteria were based on the JMHW DIC criteria [36]. The ISTH DIC criteria are used internationally, whereas both the JAAM and ISTH DIC criteria are clinically applied in the treatment of sepsis in Japan [8]. The JAAM DIC criteria have higher sensitivity than the ISTH DIC criteria, and can diagnose earlier stages of DIC [36]. Additionally, the JAAM DIC and ISTH criteria had been reported to have a high sensitivity and specificity for patient death, respectively [37].

A recent study suggested that the screening and diagnosis of DIC may reduce mortality in patients with sepsis [38], implying that interventions for coagulation abnormalities may contribute to the improvement of outcomes in patients with sepsis.

### DIC treatment

Based on the importance of coagulation disorders in the pathomechanisms of sepsis, several randomized controlled trials (RCTs) have been conducted to examine the efficacy of anticoagulant therapies, while all studies have failed to find a survival benefit of anticoagulant therapies. Based on these results, countries such as the United States and Europe do not administer anticoagulants to patients with sepsis. However, these studies did not specifically focus on patients with sepsis and DIC, but rather included the overall sepsis population. The aforementioned studies regarded physiological immunothrombosis and pathological DIC as the same entity in terms of coagulation abnormalities underlying sepsis [39]. Importantly, a meta-analysis of RCTs on the efficacy of anticoagulant therapies indicated that it showed no benefit on the survival rate in cohorts of overall sepsis and sepsis with any mildly deranged coagulofibrinolytic markers, but showed a survival benefit in the cohort with confirmed DIC [40]. These results are supported by previous post-hoc analyses of the other RCTs [41, 42]. In some countries, including Japan, DIC has been classified as a pathological coagulopathy in septic conditions; and the DIC working group of the Japanese Clinical Practice Guidelines for Management of Sepsis and Septic Shock (J-SSCG) conducted meta-analyses employing RCTs including septic patients with DIC based on this evidence and suggested antithrombin replacement therapy and administration of rhTM [8]. Meanwhile, the Sepsis Coagulopathy Asahi Recombinant LE Thrombomodulin (SCARLET) failed to determine the efficacy of rhTM in patients with sepsis-associated coagulopathy, which was defined as PT-international normalized ratio (INR) > 1.4, platelet count of 30–150 × 10^9^/L, or a decrease in platelet count > 30% within 24 h [43]. However, coagulation dysfunction defined by sepsis-associated coagulopathy may be milder than DIC, and modification of enrollment criteria was suggested immediately after the publication of SCARLET [44]. Recent studies have repeatedly focused on the importance of patient selection for anticoagulant therapies according to disease severity in addition to “sepsis with DIC” [45–49].

## TMA

TMAs are a group of disorders characterized by MAHA, thrombocytopenia, and organ damage [50, 51]. The pathological features as vascular damage include arteriolar and capillary thrombosis with abnormalities in the endothelium and vessel walls [52]. Laboratory findings that may suggest TMA include thrombocytopenia and evidence of MAHA, including decreased hemoglobin and haptoglobin levels; increased reticulocytes, lactate dehydrogenase, and total bilirubin levels; and the presence of schistocytes [50, 53]. Coagulation abnormalities, such as prolonged prothrombin time and increased fibrinogen/fibrin degradation products, are not typically observed in TMA [53]. There are several types of TMA including TTP, Shiga toxin-producing *Escherichia coli* hemolytic uremic syndrome (STEC-HUS), complement-mediated HUS (CM-HUS), secondary TMA, and as a comorbid in critical illness [54].

Factors such as medication and pregnancy can cause TMA, as in the case of secondary TMA. The treatment of TMA for these conditions involved the removal of the cause. In contrast, patients with a disintegrin-like and metalloproteinase with thrombospondin type 1 motifs 13 (ADAMTS13) deficiency or complement gene mutations may clinically manifest TMA during pregnancy, surgery, or inflammatory diseases. In this case, it is not only necessary to remove the cause but also to undergo disease-specific treatment [52].

### Thrombotic thrombocytopenic purpura

TTP is the most frequently reported disorder among TMAs. Many patients with TTP present with a triad of thrombocytopenia, microangiopathic hemolysis, and neurological abnormalities. Some patients may also present with fever and kidney abnormalities, referred to as pentads. However, neither the triad nor the pentad are reliable for diagnosing TTP. Practically, it is necessary to suspect TTP in cases of thrombocytopenia and MAHA [55].

ADAMTS13 deficiency is the main pathogenesis of TTP, which is attributed to genetic mutations and autoimmune inhibitors. TTP is diagnosed with plasma ADAMTS13 activity levels <10% [55, 56]. However, ADAMTS13 test results are not always immediately available. Thus, the PLASMIC score [57] can be easily calculated using commonly accessible findings and is useful for the risk assessment of ADAMTS13 activity <10%. It can be used as an adjunctive reference for reasonably initiating treatment while awaiting the ADAMTS13 test results. TTP is classified as congenital/hereditary or acquired/immune. Congenital TTP can develop into adulthood. Although ADAMTS13 activity is persistently low, some patients remain asymptomatic until a trigger precipitates symptoms [55].

The treatment of immune TTP typically involves plasma exchange (PEX) to remove ADAMTS13 inhibitors and supplement normal plasma [58]. In addition to PEX, glucocorticoids, rituximab, caplacizumab, and recombinant ADAMTS13 ( currently in phase 3 clinical trials) are considered treatment options for TTP [56]. Rituximab is a monoclonal antibody against CD20 that prevents immune TTP relapse [59]. Caplacizumab is a monoclonal antibody fragment that targets the A1 domain of vWF, promptly blocks platelet-vWF interactions, and prevents microvascular thrombosis in the arterioles and capillaries. The optimal clinical use of caplacizumab is yet to be determined; however, it is likely to be highly effective when administered early in the disease [59]. The treatment options for hereditary TTP include plasma infusion or recombinant ADAMTS13, a drug approved by the Food and Drug Administration in late 2023. In phase 3 and 3b studies, treatment with recombinant ADAMTS13 rapidly resolved the acute TTP events and increased the platelet counts in patients with congenital TTP without causing serious adverse events [60]. This is useful as it can supplement the deficient enzyme without exposing the patient to donor plasma [61].

### Shiga toxin-producing *Escherichia coli* hemolytic-uremic syndrome (STEC-HUS)

HUS can be classified into two types: typical HUS, which is accompanied by diarrhea and atypical HUS, which is not. Most typical HUS cases are caused by Shiga toxin-producing *Escherichia coli* (STEC) bacteria, with *E. coli* O157:H7 being the most common and predominant pathogen worldwide [62]. Shiga toxin can directly damage the endothelium due to its prothrombotic effects and stimulation of endothelial cell release by ultralarge von Willebrand multimers. Consequently, this reaction activates platelets, leading to aggregation and occlusion of the microvasculature [50]. STEC-HUS is the most frequent type of TMA and is most commonly observed in children under 5 years of age, although it can also occur in adults [50], Additionally, 15% of people with symptoms of STEC infection develop HUS [62]. Symptoms of STEC infection, including diarrhea, abdominal pain, fever, and vomiting, typically appear 2–12 days (median, 3 days) after bacterial ingestion. At symptom onset, laboratory test results for TMA are usually negative [62]. Renal damage is common in patients with STEC-HUS. Severe cases may also present with neurological symptoms, such as mental disturbances, seizures, and coma [63].

A stool sample is required to test for STEC infection. Rectal swabs were used if stool samples were unavailable. Sorbitol-MacConkey agar is a type of media used to isolate *E. coli* O157:H7 [64]. However, the results of stool culture tests may be unreliable as the bacteria may only be present in the stool for a few days. Antibodies against STEC lipopolysaccharides in the serum can persist for several weeks and may help diagnose STEC infection [65].

Treatment for STEC-HUS involves managing of symptoms, such as hemodynamic and renal management. Moreover, approximately 60% of patients require dialysis during the acute phase [66]. Antibiotics are thought to increase the risk of HUS and are, not recommended for use in STEC infections. However, research on this topic is mixed, with some studies suggesting that antibiotics increase the risk of HUS, while others found no effect of HUS with antibiotic use [67]. There is no clear evidence that PEX is beneficial for the treatment of STEC-HUS [62]. The American Society for Apheresis advises that the role of PEX in STEC-HUS treatment is uncertain, and the decision to use PEX should be made on a case-by-case basis [50, 68]. However, it can be difficult to distinguish STEC-HUS from other types of TMA that may respond to PEX, such as CM-HUS and TTP, as they manifest similar gastrointestinal symptoms. Thus, PEX may be used in some patients with STEC-HUS.

### Complement-mediated hemolytic uremic syndrome (CM-HUS)

Atypical HUS (aHUS) does not cause diarrhea. It is often caused by genetic or acquired defects in the regulation of complement activation, known as complement-mediated HUS (CM-HUS) [69, 70]. CM-HUS can occur in children and adults. It is caused by mutations in complement control proteins (such as Factor H, Factor I, membrane co-factor protein (MCP), and thrombomodulin (THBD)), gain-of-function mutations that make C3 and Factor B less susceptible to inactivation, or antibodies that attack specific complement components (such as Factor H). Uncontrolled complement activation results in the activation of platelets and microvascular endothelial injury, leading to widespread microthrombosis and the clinical phenotype of CM-HUS [71].

Organ damage is often characterized by significant kidney injury. Clinical manifestations of CM-HUS include the common triad of clinical features of TMAs as well as extrarenal symptoms, including neurologic, cardiovascular, and gastrointestinal involvement. The neurological symptoms are the most common non-renal manifestations, occurring in 8-48% of cases. These symptoms include seizures, vision loss, hemiparesis, headaches, altered consciousness, and hallucinations [72]. Gastrointestinal complications, including diarrhea, are common in CM-HUS. Diarrhea is classically associated with STEC-HUS but is also observed in approximately 50% of patients with CM-HUS [72]. In CM-HUS, triggering events (such as diarrhea, upper respiratory tract infections, and pregnancy) have been documented in 39-70% of cases. These events are often considered as triggers rather than causes of the disease [73, 74].

Certain genetic mutations or autoantibodies against Factor H may be used to diagnose CM-HUS. However, these tests may not be useful for making acute treatment decisions, as test results may not be available for months, and mutations are only found in about half of the patients diagnosed with CM-HUS [71].

Eculizumab, a humanized monoclonal antibody, targets the complement component C5 to stop abnormal activation of the complement pathway, decreasing damage to the endothelium and thus the organ dysfunction [75]. Recently, ravulizumab was reported to be non-inferior to eculizumab, with the added benefit of less frequent administration. These may be superior to plasma therapy for the treatment of CM-HUS because they can directly turn off abnormal complement activity [75, 76]. PEX removes autoantibodies or mutated circulating complement regulators and replaces defective complement regulators. PEX is accepted as the first-line therapy for cases caused by anti-Factor H antibodies; however, its optimal role in cases with genetic abnormalities has not been established. Moreover, PEX is recommended for patients with CM-HUS while the type of TMA is being investigated [68]. Hemodialysis is also required for severe acute kidney injury, which often leads to end-stage renal disease in patients with CM-HUS. Early initiation of eculizumab therapy is associated with greater improvement in estimated glomerular filtration rate, suggesting that timely diagnosis and treatment with eculizumab are important for patient outcomes [71, 72, 77].

### Secondary TMA

Secondary TMA is a broad concept that has not yet been well-defined. Several factors may contribute to the development of TMA [78]. We focused on common etiologies of secondary TMA, including drugs, pregnancy, and cancer [54, 78, 79].

#### Drug

Drug-induced TMA (DI-TMA) is caused by immune-mediated and direct toxicity [78]. In immune-mediated DI-TMA, the drug triggers the formation of antibodies against various cells, causing damage to the endothelium and platelet consumption [54]. Examples of drugs that can cause this type of DI-TMA include quinine, which causes systemic symptoms and acute kidney injury upon first exposure to the drug [80]. Meanwhile, decreased expression of VEGF may play a role in the direct toxicity of DI-TMA [54]. Cyclosporine, tacrolimus, and sirolimus are known to cause this type of DI-TMA, which can occur either acutely or after long-term exposure [80]. The main treatment for DI-TMA is removal of the offending drug and provision of supportive care [54].

#### Pregnancy

Hemolysis, elevated liver enzyme levels, and low platelet count (HELLP) are part of the spectrum of preeclampsia (a condition characterized by proteinuria and hypertension) and can lead to serious maternal and neonatal morbidity and even mortality. HELLP is characterized by microangiopathic disease as evidenced by schistocytes in the peripheral smear and a negative Coombs test result. Distinguishing HELLP from pregnancy-triggered TTP or CM-HUS can be difficult. TTP or CM-HUS should be proactively considered when HELLP does not improve after prompt blood pressure control and delivery [54].

#### Cancer

TMA is commonly associated with two types of cancers, gastric and breast cancers, specifically mucin-producing adenocarcinomas [54]. It has been hypothesized that mucin may have a direct effect on the endothelium, perturbing the production and release of vWF. Additionally, TMA may be caused or exacerbated by direct contact between erythrocytes and circulating cancer cells and the presence of tumor emboli within small blood vessels [81]. Physicians should consider both cancer-related and chemotherapy-induced TMA in patients with cancer who present with TMA. Although up to 90% of the patients with cancer-related TMA have metastatic disease, chemotherapy-induced TMA often has minimal or no detectable malignancy. Chemotherapeutic agents, such as mytomycin, gemcitabine, and VEGF pathway inhibitors, are commonly associated with chemotherapy-induced TMA. It is important to monitor patients receiving medications associated with TMA for signs of hypertension, hematuria/proteinuria, and decreased kidney function because the classic triad of TMA symptoms may not always be present. Treatment typically involves discontinuation of the causative agent and effective blood pressure control with renin-angiotensin blockers [81].

## Other critical thrombocytopenic diseases

In addition to DIC and TMA, multiple severe conditions associated with sepsis are linked with thrombocytopenia, such as haemophagocytic lymphohistiocytosis (HLH). HLH, including hemophagocytic syndrome, is a severe hyperinflammatory syndrome driven by activated macrophages and T cells due to the impairment of natural killer and cytotoxic T cells; it causes cytopenia and organ dysfunction due to an extreme inflammatory cytokine storm and tissue damage, resulting in high mortality [82–86]. HLH is diagnosed according to the HLH-2004 diagnostic criteria based on the following eight parameters: fever, splenomegaly, cytopenia, hypertriglyceridemia and/or hypofibrinogenemia, hemophagocytosis, low/absent natural killer cell activity, hyperferritinemia, and high soluble interleukin-2-receptor levels [87, 88]. Infections are the most prevalent triggers of HLH. Viral infections, such as the Epstein-Barr virus, are the most frequent triggers, and bacterial infections also induce HLH [82]. HLH is an important differential diagnosis in critically ill patients with sepsis, especially in those not responding to sepsis treatment [87, 88]. Intravascular lymphomas (IVLs) can trigger HLH. An IVL is a rare type of non-Hodgkin lymphoma characterized by an intravascular proliferation of malignant lymphocytes, an aggressive clinical course, and a short prognosis [89]. The Asian variant of IVL presents a higher incidence of thrombocytopenia, with the etiology not solely attributed to HLH as a clinical manifestation (76% vs. 29%), compared with the variant prevalent in Western countries [90]. As it mimics sepsis, carefully distinguishing IVLs from septic DIC is necessary.

COVID-19, caused by severe acute respiratory syndrome coronavirus 2 (SARS-CoV-2), induces thrombocytopathy, characterized by platelet hyperactivation, thrombocytopenia, and impaired platelet reactivity. Thrombocytopathy is a prominent feature of COVID-19, contributing to excessive thrombus formation via platelet hyperactivation and augmented reactivity or heightened bleeding tendencies due to thrombocytopenia [91–98]. Both venous and arterial thrombotic complications are commonly observed in patients with COVID-19, which is characterized by elevated D-dimer levels [99–103]. COVID-19-associated coagulopathy (CAC) is characterized by a prothrombotic state caused by inflammation, thrombocytopenia, coagulation activation, and endotheliopathy [93–96, 98]. CAC shares common features with sepsis-induced coagulopathy (SIC) and DIC, although some differences were noted between the two pathophysiologies. Excessive thrombin generation, a manifestation of DIC, has not been conclusively established. Additionally, decreased platelet count and prolonged prothrombin time are uncommon or tend to be mild in patients with COVID-19, in contrast to SIC and DIC [104–107]. These coagulofibrinolytic marker manifestations contradict the diagnoses of SIC and DIC. Iba et al. proposed diagnostic criteria specific for CAC, which are distinctly different from those for SIC and DIC [104]. Patients with COVID-19 should be diagnosed with CAC when they meet two or more of the following criteria: (1) a decrease in platelet count (less than 150×109/L), (2) an increase in D-dimer levels (more than two times the upper limit of normal), (3) a prothrombin time of >1 s or an INR of >1.2, (4) a decrease in fibrinogen levels, and (5) the presence of thrombosis. Heparin may protect organ function by exerting antithrombotic, anti-inflammatory, and antiviral effects against SARS-CoV-2 infection, and many studies have demonstrated the benefits of heparin use in patients with COVID-19 [108, 109].

Furthermore, thrombocytopenia is a common complication of liver diseases, including cirrhosis. Thrombocytopenia is a common complication of chronic liver diseases such as liver cirrhosis. Multifactorial mechanisms contribute to the development of thrombocytopenia, including platelet sequestration commonly accompanied by hypersplenism and portal hypertension, viral-induced bone marrow suppression, autoantibodies, and decreased thrombopoietin (TPO) production [110–112]. Acute-on-chronic liver failure, a syndrome characterized by the acute deterioration of preexisting chronic liver disease, can occur as a result of bacterial infection, leading to multiple organ failure and high mortality [113, 114]. Bacterial infection also causes sepsis-associated liver dysfunction including hypoxic hepatitis, cholestasis, hepatocellular injury, and sclerosing cholangitis [115, 116]. Protein synthesis dysfunction due to acute or acute chronic liver failure can cause coagulopathy. Whether liver dysfunction itself can lead to DIC has been debated; however, DIC can easily develop in patients with liver failure triggered by bacterial infections [115, 117, 118]. In patients with liver failure, it is necessary to differentiate DIC or detect its concurrence by assessing the coagulofibrinolytic activity.

Additionally, when considering complications arising from the treatment of critically ill patients, there is a condition called Heparin-Induced Thrombocytopenia (HIT) that leads to thrombocytopenia. Platelet factor 4 (PF4) stored in platelet α-granules is released at the sites of platelet activation and forms PF4-heparin complexes upon administration of heparin. B lymphocytes recognize PF4-heparin complexes and generate antibodies against these complexes (HIT antibodies) to form immunocomplexes. These immunocomplexes bind and activate platelets and monocytes via FcγRIIa receptors, followed by thrombin generation due to expression of tissue factor and microparticles [119, 120]. The clinical manifestations of thrombocytopenia and thrombosis develop 5–14 days after heparin exposure. Platelet count often shows a 30–50% decline from baseline, which is typically not associated with bleeding tendency. Thrombotic complications, such as deep venous thrombosis, pulmonary embolism, and catheter-related thrombosis, predominantly develop in the veins rather than in the arteries [119–121]. The 4Ts clinical scoring system, which evaluates four features, thrombocytopenia, thrombosis, timing of onset, and exclusion of other causes, is widely used to predict HIT [122]. HIT-mimicking disorders without proximate heparin exposure have also been reported after infection and other conditions, such as “spontaneous HIT.” PF4 plays an important role in innate immunity and inflammatory responses and can interact with many polyanions, such as lipopolysaccharides and DNA [123, 124].

## Relationship between DIC and TMA

### Differences and similarities between DIC and TMA

Both DIC and TMA cause vascular endothelial cell injury and microvascular thrombosis, which is caused by the activation of coagulation in DIC and platelets in TMA. In other words, fibrin thrombosis occurs on the venous side of the microvessels in DIC, while platelet thrombosis occurs on the arterial side of the microvessels in TMA [6]. Thrombosis confirmed using TMA is associated with high blood pressure, which may be caused by acute kidney injury, a frequently observed organ dysfunction [125]. In contrast, patients with DIC often suffer from hypotension as a circulatory dysfunction, which is often complicated by respiratory failure.

Regarding laboratory data, prolonged PT and marked elevations in fibrin-related markers, such as soluble fibrin monomers, FDP, and D-dimer, were observed in most DIC cases, but not in TMA. In addition to thrombocytopenia, increased levels of total bilirubin and LDH caused by the development of MAHA were confirmed in patients with TMA. Importantly, DIC is diagnosed by universal coagulation scoring, whereas most patients with TMA can also be diagnosed with DIC using the DIC diagnostic criteria. Conversely, approximately 10% of patients with DIC are diagnosed with TMA [6]. Since there are specific therapies such as plasma exchange for TTP and anti-complement therapy for CM-HUS, as well as anticoagulant therapies for DIC, early differential diagnosis between DIC and TMA is important; however, DIC and TMA can coexist, and differentiating between DIC and TMA is “easier said than done”.

### Differences in algorithms to differentiate sepsis-related DIC from TMA based on the disease concept of DIC

In an editorial on ICU patients with thrombocytopenia, Vincent et al. published an algorithm to rapidly differentiate DIC from TMA [11]. This algorithm first checks for the presence of MAHA and then evaluates the coagulation profile, including PT, APTT, and FDP, to differentiate DIC in patients with thrombocytopenia. Behind this diagnostic approach, we can see that DIC is not considered a treatment target and should be more focused on treating the underlying disease. In contrast, another algorithm presented by the J-SSCG2020 working group for DIC estimates coagulation abnormalities by evaluating PT or FDP to diagnose DIC, and then checks for MAHA after DIC is ruled out [126]. This initial DIC diagnosis in patients with thrombocytopenia is based on the much higher prevalence of DIC than that of TMA and the recognition of the importance of early diagnosis and treatment of DIC in Japan. The ISTH guidelines also proposed an algorithm similar to that of the J-SSCG2020, which first evaluates coagulation abnormalities [127].

## Comorbidity of DIC and TMA

In thrombocytopenia and organ dysfunction, DIC is more frequent than in TMA. As mentioned previously, early differentiation and treatment are important for DIC and TMA because their treatment methods are different. DIC and TMA have similarities; however, all coagulation, fibrinolysis, and platelet systems are activated in DIC, and only platelets are markedly activated in TMA. However, clinicians must always consider cases in which septic DIC and TMA are comorbid [6]. Crosstalk between the complement and coagulation systems is crucial in TMA and DIC. Thus, the comorbidities of DIC and TMA have been reported. Here, we describe the association between DIC and TMA secondary to infection and/or sepsis.

### Comorbidity of DIC and aHUS

#### DIC and CM-HUS

The dysregulation of the alternative complement pathway causes endothelial cell damage. Complement activation in patients with DIC is also well known; therefore, crosstalk between the complement and coagulation systems may be important in these patients.

Abe et al. reported a case of CM-HUS complicated by septic DIC [128]. Sepsis treatment only improved DIC, and TMA features became more prominent. Plasma exchange followed by eculizumab administration improved the TMA conditions. Genetic testing suggests that a mutation in the CFH gene may have been involved. In this case, the authors concluded that septic-DIC triggered abnormal complement activation, leading to CM-HUS.

There are some reports on the frequency of the coexistence of DIC and CM-HUS. Fujisawa et al. conducted a nationwide study of patients clinically diagnosed with CM-HUS [129]. In this study, 118 patients with CM-HUS in Japan were enrolled between 1988 and 2016. The coagulation-fibrinolysis system was evaluated by PT-INR, APTT, fibrinogen, and FDP values at the initial visit. Most patients clinically diagnosed with CM-HUS did not meet the DIC criteria. In contrast, Sakurai et al. reported 3 out of 15 (20%) CM-HUS patients fulfilled the diagnostic criteria for DIC by the JSTH [130]. Although these patients were classified as having the basic type, a frequency of 20% was noteworthy. In addition, the authors mentioned that the distribution of DIC scores in patients with CM-HUS indicated that they might sequentially progress to DIC. In a previous nationwide study, 75% of patients had an infection as a probable trigger for CM-HUS [129]. The possibility of DIC following CM-HUS should be considered depending on the management of the infection that triggers CM-HUS.

#### DIC and coagulation-mediated TMA

Thrombomodulin activates protein C and triggers the anticoagulation system. In addition, thrombomodulin activates TAFI to degrade anaphylatoxins C3a and C5a and acts as a co-factor of CFI-mediated C3b cleavage in the alternative pathway. Therefore, THBD may be associated with the coagulation and complement pathways. According to Japanese guidelines [131], THBD mutations are classified as causes of coagulation-mediated TMA.

In the first reported international registry, THBD mutations were identified in 7 of 152 (4.6%) patients with aHUS [132], a type of HUS that does not involve STEC. The condition associated with this THBD mutation is clinically aHUS, with a reported frequency of 3–5% [133–135]. Interestingly, only 1 in 104 (1%) patients with aHUS was identified to have a THBD variant combined with another variant in Japan [129]. Thus, the mechanisms underlying the development of aHUS, including THBD mutations, are not fully understood.

The involvement of thrombomodulin in the development of TMA and its possible role in controlling its pathogenesis are remarkably interesting. Suppression of thrombomodulin expression due to sepsis or a systemic inflammatory response may trigger TMA. The effect of thrombomodulin on transplant-related TMA has also been previously reported [136]. Further research on the etiology and therapeutic potential of thrombomodulin in TMA is required.

### Comorbidity of DIC and secondary TMA

Systemic inflammatory responses, such as sepsis, pancreatitis, and trauma, decrease ADAMTS13 activity. The degree of the decline in ADAMTS13 activity varies, with some cases decreasing to below 10%, which is the diagnostic criterion for TTP. Lower ADAMTS13 activity due to sepsis is also associated with a poor prognosis [137].

The relationship between ADAMTS13 activity and organ failure has been reported in patients with septic DIC [138]. This study showed that the distribution of ADAMTS13 activity in patients with septic DIC varied widely and that 17 of 109 (15.6%) patients had extremely low (< 5%) ADAMTS13 activity. Furthermore, ADAMTS13 activity of < 20% in patients with septic DIC is associated with renal dysfunction. In another study, patients with DIC showed 60.1% ADAMTS13 activity, which was lower than that observed in patients without DIC [139]. In this study, only approximately 20% of the patients had infection as the cause of DIC, which may explain why the distribution of ADAMTS13 differed from that of septic DIC described above. Interestingly, low ADAMTS13 levels (< 56.4%) were associated with high mortality, with or without DIC. Habe et al. revealed that ADAMTS13 activity was significantly decreased in any of the patients with DIC (35.0, 19.5-51.6%), non-DIC (63.7,47.5-85.0%), TTP (undetectable, undetectable-6.3%), and aHUS (50.0,36.6-73.1%) compared to those in healthy volunteers (111.0,95.0-125.0%) (data are shown as medians,15-75 percentile). DIC patients with infectious disease showed significantly lower ADAMTS13 activity (30.0, 15.9-49.4%) than non-DIC patients with infectious disease (50.7, 36.3-68.8). DIC patients with infectious diseases also show a high vWF propeptide level, which is associated with a low survival rate [140]. In the retrospective analysis, 5 of 20 (20%) secondary TMA patients fulfilled the JSTH-DIC criteria, and two of the patients were classified into the infectious type. The results also indicate that the coexistence of DIC and TMA occurred at a certain frequency [130]. ADAMTS13 activity was decreased in these patients (45, 37.4-62.4%; median, interquartile range).

Several case reports have described cases of DIC and secondary TMA. One case report showed that *Capnocytophaga canimorsus* sepsis causes DIC and secondary TMA. ADAMTS13 activity on the day of admission was detected on the fifth hospital day and decreased by < 1%. Notably, no schistocytes were observed in the blood smear on the day of admission, despite the other characteristics of TMA. The patient’s condition improved after administration of fresh frozen plasma [141]. In another case of *Capnocytophaga canimorsus* infection followed by DIC and secondary TMA, the activity of ADAMTS13 was not reduced (77.2%). Plasma exchange started on day 5 and the patient’s condition improved [142]. Sakamaki et al. reported a case of septic DIC complicated with TMA. The patient was treated for DIC due to urinary tract infection; however, despite improvement in the coagulation system, neurological symptoms and renal dysfunction persisted. The patient was considered for TMA and underwent plasma exchange. At this point, the ADAMTS13 activity was 44% without the ADAMTS13 inhibitor. The TMA improved with plasma exchange, except for renal function, and the patient required maintenance dialysis. In this case, plasma exchange was initiated on day 7; however, elevated LDH and AKI levels were present, suggesting that TMA may have developed earlier [143].

A variety of pathogenic microorganisms are known to cause TMA, including Shiga toxin-producing *E. coli*, *Streptococcus pneumoniae*, parvovirus B19, and severe acute respiratory syndrome coronavirus type 2. The pathogenesis of infection-induced TMA is complex and includes direct endothelial injury, ADAMTS13 inhibition, complement activation, or a combination of these mechanisms [144]. Even when the diagnostic criteria for DIC are met, the coexistence or subsequent development of TMA should be noted when sepsis or a systemic inflammatory response exists.

## Practical approach to thrombocytopenia in patients with sepsis

### A novel diagnosis and treatment flow for patients with thrombocytopenia associated with sepsis

**Fig. 1:**
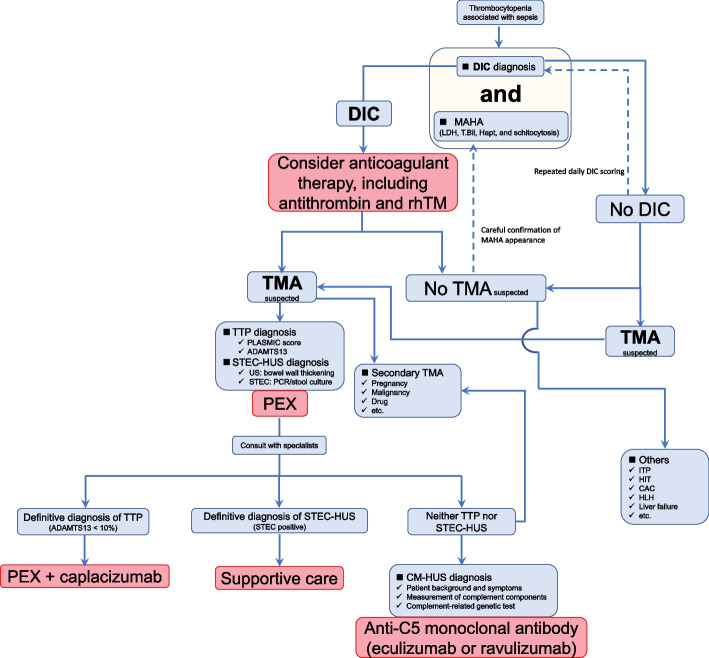
A novel diagnosis and treatment flow for patients with thrombocytopenia associated with sepsis. Patients with sepsis-associated thrombocytopenia were checked for MAHA in the presence of TMA, in parallel with DIC diagnosis using the DIC scoring system. If TMA is suspected, a definitive diagnosis of HUS and TTP is made by confirmation of the Shiga toxin-producing ability of *E. coli* isolated from the patient’s stool and ADAMTS13 activity of < 10%, respectively, and PEX should be initiated. When TMA is suspected, secondary TMA may be diagnosed on the basis of the patient’s background. If neither HUS nor TTP is diagnosed, the possibility of CM-HUS should be considered. It is important to decide whether to administer an anti-C5 monoclonal antibody based on the patient’s medical condition before the results of genetic testing are known. If there are no signs of MAHA and TMA appears negative, differentiation of HIT, ITP, etc., should be performed with careful examination of the MAHA appearance. ADAMTS13, a disintegrin-like and metalloproteinase with thrombospondin type 1 motifs 13; CAC, COVID-19-associated coagulopathy; CM-HUS, complement-mediated hemolytic uremic syndrome; DIC, disseminated intravascular coagulation; hapt, haptoglobin; HIT, heparin-induced thrombocytopenia; HLH, hemophagocytic lymphohistiocytosis; ITP, Immune thrombocytopenia; MAMA, microangiopathic hemolytic anemia; PCR, polymerase chain reaction; PEX, plasma exchange; rhTM, recombinant human thrombomodulin; TMA, thrombotic microangiopathy; TTP, thrombotic thrombocytopenic purpura; US, ultrasound

Previous studies indicated that the prevalence of TMA is remarkably lower than that of DIC. Although interest in TMA in the field of intensive care has increased over the last decade, its epidemiology has not yet been fully evaluated. This means that due to adherence to the DIC diagnosis caused by the known high prevalence rate of DIC, some patients may be undiagnosed with TMA, resulting in mortality or severe complications. To reduce the number of such cases, we developed a novel diagnostic and treatment flow for patients with thrombocytopenia associated with sepsis (Fig. 1). The differences between this flow and those previously published [53, 127], as mentioned above (Section 6.2), are as follows: i) simultaneous implementation of DIC diagnosis and confirmation of MAHA for TMA diagnosis, ii) incorporation of specific treatment methods in the flow, and iii) longitudinal diagnosis and treatment flow of TMA with real clinical timeframes. Based on the J-SSCG 2020, the JAAM DIC scoring system [11, 145] was used for DIC diagnosis in consideration of early diagnosis and initiation of treatment. Once DIC is diagnosed (JAAM score ≥ 4), anticoagulant therapy should be considered in addition to the treatment of the underlying disease. Importantly, at the same time as the DIC diagnosis, the presence of MAHA should be confirmed to diagnose TMA. General blood test parameters such as LDH and T-bil were first checked and, if suspected, haptoglobin levels and/or the presence of schistocytes were confirmed. If TMA was suspected, the next step was to diagnose TTP and HUS. A definitive diagnosis of TTP and HUS requires ADAMTS13 activity < 10% and confirmation of the Shiga toxin-producing ability of *E. coli* isolated from the patient’s stool, respectively. However, it may be important to not hesitate in initiating PEX according to clinical symptoms as these test results are not immediately available. Consultation with specialists, such as hematologists or nephrologists, should also be considered. When TMA is suspected, secondary TMA may be diagnosed on the basis of the patient’s background. In such cases, supportive therapy and treatment for the underlying primary disease should be provided. If ADAMTS13 activity is < 10%, TTP is diagnosed and administration of caplacizumab is considered in addition to PEX. If HUS is diagnosed, supportive care would be continued until the patient condition improves. If neither HUS nor TTP is diagnosed, the possibility of secondary TMA should be considered again, as well as CM-HUS. Genetic testing is required for a definitive diagnosis of aHUS. It is sometimes necessary to proceed with the administration of anti-C5 monoclonal antibodies, including eculizumab and ravulizumab, based on the patient ’s medical condition before the results of genetic testing are known, as it takes months to obtain the results. If there are no signs of MAHA and TMA appears negative, differentiation of HIT, and ITP, should be performed with careful examination of the MAHA appearance.

### Future perspective

The diagnosis and treatment flow were developed by the J-STAD study group, which consisted of experts in sepsis care in Japan. A prospective study with the cooperation of members of the J-STAD study group will be conducted to validate the clinical significance of this flow. We believe that this study will help accumulate evidence on the epidemiology, clinical course of TMA, and efficacy of new specific therapeutic agents, including eculizumab and ravulizumab for CM-HUS and caplacizumab for TTP, consequently improving outcomes in patients with thrombocytopenia associated with sepsis.

## Conclusions

Amidst a high incidence rate of DIC, a sepsis patients with thrombocytopenia may consequently be undiagnosed with TMA. Sepsis may complicate the diagnosis as it can cause DIC and trigger TMA. To reduce overlooked of TMA cases, we developed a new diagnostic and treatment flow for patients with sepsis-associated thrombocytopenia. Our flow simultaneously diagnoses DIC, confirms MAHA for TMA diagnosis, incorporates specific treatment methods for TMA, and considers real clinical timeframes for the longitudinal diagnosis and treatment of TMA. This new diagnostic and treatment flow envisioned to help patients with TMA to receive appropriate treatment and alleviate their conditions.

## Data Availability

No datasets were generated or analysed during the current study.

## Abbreviations

ADAMTS13: A disintegrin-like and metalloproteinase with thrombospondin type 1 motifs 13
AIPF: Acute infectious purpura fulminans
APS/CAPS: Antiphospholipid syndrome/catastrophic APS
CAC: COVID-19-associated coagulopathy
CM-HUS: Complement-mediated hemolytic uremic syndrome
DIC: Disseminated intravascular coagulation
FDP: Fibrin degradation product
HIT: Heparin-induced thrombocytopenia
HLH: Hemophagocytic lymphohistiocytosis
HUS: Hemolytic uremic syndrome
ISTH: International Society on Thrombosis and Hemostasis
ITP: Immune thrombocytopenia
JAAM: Japanese Association of Acute Medicine
JMHW: Japanese Ministry of Health and Welfare
JSTH: Japanese Society on Thrombosis and Hemostasis
MAHA: Microangiopathic hemolytic anemia
PCR: Polymerase chain reaction
PEX: Plasma exchange
PT-INR: Prothrombin time-international normalized ratio
PT: Prothrombin time
rhTM: Recombinant human thrombomodulin
SIRS: Systemic Inflammatory Response Syndrome
STFS: Severe fever and thrombocytopenia syndrome
TAT: Thrombin-antithrombin complex
TMA: Thrombotic microangiopathy
TTP: Thrombotic thrombocytopenic purpura
